# Physicochemical Process, Crustacean, and* Microcystis* Biomass Changes In Situ Enclosure after Introduction of Silver Carp at Meiliang Bay, Lake Taihu

**DOI:** 10.1155/2017/9643234

**Published:** 2017-01-05

**Authors:** Chengjie Yin, Longgen Guo, Chunlong Yi, Congqiang Luo, Leyi Ni

**Affiliations:** ^1^Donghu Experimental Station of Lake Ecosystems, State Key Laboratory for Freshwater Ecology and Biotechnology of China, Institute of Hydrobiology, Chinese Academy of Sciences, Wuhan 430072, China; ^2^University of Chinese Academy of Sciences, Beijing 100049, China; ^3^Collaborative Innovation Center for Efficient and Health Production of Fisheries in Hunan Province, Key Laboratory of Health Aquaculture and Product Processing in Dongting Lake Area of Hunan Province, Hunan University of Arts and Science, Changde 415000, China

## Abstract

In order to control cyanobacteria blooms with silver carp in Lake Taihu, an in situ experiment was carried out by stocking silver carp at a biomass of 35, 70, and 150 g m^−3^ and no carp control in waterproof enclosures. Physicochemical water parameters and biomass of plankton were measured in enclosures to evaluate the suitable stocking density of silver carp for relieving internal nutrients and constraining cyanobacteria growth in Lake Taihu. It is found that the 35 g m^−3^ silver carp group and 70 g m^−3^ silver carp group presented lower total phosphorus, lower chlorophyll-*a*, and higher water transparency. Increased nitrogen to phosphorus ratio, which indicated the result of algae decline in fish presence enclosures, was attributed to decline of phosphorus. Phosphorus decline also exerted limitation on reestablish of cyanobacteria bloom. Crustacean zooplankton biomass and* Microcystis* biomass decreased significantly in fish presence enclosures. Silver carp could be more effective to regulate algae bloom in enclosures with dense cyanobacteria. Therefore, nonclassic manipulation is supposed to be appropriate method to get rid of cyanobacteria blooms in Lake Taihu by stocking 35 to 70 g m^−3^ silver carp in application.

## 1. Introduction

In freshwater ecosystems, fish affect the structure and dynamics of pelagic plankton communities by trophic cascading effects [1, 2], which usually relates consumers to their environments by food web chains [3]. Removal of planktivorous fish could relieve the predation pressure on zooplankton community by top-down control; thus the enhancement of crustaceans zooplankton leads to decline of algal density [4, 5]. This method, used to be defined as classic biomanipulation, however, usually malfunctions [6] because the presence of zooplankton grazing-resistant species such as frequent carpet of fetid cyanobacteria disables or weakens top-down force in nutrient enrichment lakes. Moreover, as the absence of large size zooplankton such as* Daphnia* in these lakes, crustacean communities are not able to control algal bloom by zooplankton-target manipulation: indeed grazing pressure by zooplankton is useless [7].

Introduction of filter-feeding planktivorous fish to hypertrophic shallow freshwater lakes successfully is another effective method to regulate the algal community which is called nonclassic biomanipulation [8–10]. As for nonclassic biomanipulation, planktivorous fish directly collect food by filtering water via their gill rakers and hence, unselectively ingest plankton and detritus. However, the ecological effects of filter-feeding fish introduced to specific lakes have remained controversial [5]. Three conditions for successful control of algae by planktivorous fish's grazing should be taken into account: (1) the stocking density and body size of filter-feeding silver carp [5]; (2) the initial plankton species pool [11] and (3) environmental conditions [12].

Lake Taihu is the third largest shallow freshwater lake located at the lower reach of the Yangtze River. Due to accumulative nutrient-rich sewage and agricultural run-off inflow, it became eutrophic with heavy cyanobacteria blooms from late spring to autumn every year in last decades [13]. In this lake, dominant crustacean zooplankton has changed from large body sized individuals, such as* Daphnia*, to small ones, such as* Limnoithona sinensis* during the last 60 years [14]. Hence, biomanipulation method to control algal blooms to improve water quality became impossible in Lake Taihu. As for nonclassic biomanipulation successfully in the hypereutrophic Lake Donghu, we consider using filter-feeding planktivorous fish to control cyanobacteria blooms in situ enclosure experiments in Lake Taihu.

Some previous studies [9, 10] investigated the ecological and biological effects of planktivorous silver carp and bighead carp with a certain density in large pen/enclosure at Meiliang Bay and Gonghu Bay, which are all located at the most serious bloom area in Lake Taihu. But we could not obtain optional stocking density of planktivorous fishes to control the dense algal blooms in this lake. Therefore, we carried out in situ enclosure experiments to explore the suitable stocking density of filter-feeding silver carp on controlling cyanobacteria blooms and hope to enhance fisheries resources for sustainable development based on decreasing the algal blooms and reasonable fish biodiversity in future.

## 2. Materials and Methods

### 2.1. Experiment Site and Device Settlement

**Figure 1:**
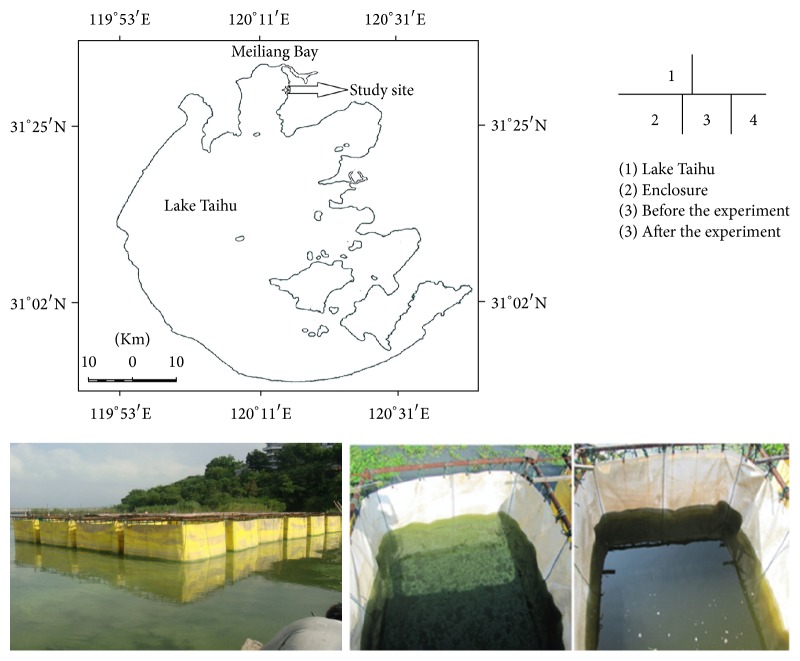
The study site and experimental enclosures.

Our experiment was located at Meiliang Bay (31°31–325′N, 120°09–340′E, Figure 1), which is administrated by Wuxi City, Jiangsu Province (Figure 1). Meiliang Bay receives heavy nutrient loads and has suffered eutrophication with serious algal blooms. The mean total phosphorus (TP) and total nitrogen (TN) are 0.1 mg L^−1^ (max 0.2 mg L^−1^) and 2.3 mg L^−1^ (max 5.6 mg L^−1^) in the northern east of Meiliang Bay [9, 15].

Four facets cuboid waterproof PVC enclosures (2.5 × 2.5 × 3 m installed in water depth at approximate 1.5 m littoral zone) were fixed to a cage of steel pipes. Bottom margins of each enclosure with heavy stone cages were sank into the lake sediment as possible to avoid water exchange between inside and outside of the enclosure during the study period. Its cost is about 3000 YUAN (equal to about 450 USD) per enclosure including all the materials and labour costs.

### 2.2. Study Protocol and Sampling Collection

Planktivorous fish silver carp (average weight 137 g ± 3 g) were obtained from a local aquatic farm and then acclimated in a nearby pond until they were transferred into the enclosures. The whole experiment aimed to explore the proper density of silver carp to control algae bloom.

In this experiment, 12 enclosures were chosen and randomly divided into four groups with triplications representing for control group (CG), low fish density group (LDG), medium fish density group (MDG), and high density group (HDG). CG, LDG, MDG, and HDG had fish biomass at 0, 35, 70, and 150 g m^−3^. The experiment lasted from 30th May to 23rd June 2011 with a sampling interval of 3-4 days depending on the local weather. The average cyanobacteria biomass before the experiment in each enclosure was approximate about 5.8 g L^−1^ before the fish were introduced (confirmed with 2010* Microcystis* biomass).

### 2.3. Experimental Parameters

Integrated water sample was collected by a 5 liter modified Patalas's bottle sampler. We determined water temperature, dissolved oxygen (DO), total dissolved solids (TDS), and pH using YSI Professional Plus (YSI Inc., Yellow Springs, Ohio, USA) water quality monitor. Water transparency was determined by using a 20-cm in diameter Secchi's disk and represented as Secchi's depth (*Z*_Sd_). Turbidity was measured by a turbidimeter (Model TN-100, Eutech Instrument, Pte Ltd., Singapore).

50 mL samples of quantitative crustaceans were collected by filtering 10 L integrated water samples through 25^#^ (69 *μ*m) plankton net and then fixed with 1 mL saturated formalin. All individuals were counted after precipitation for 1 day by using an Olympus compound microscope (model BH2-RFC; Olympus America, Inc., Melville, NY, USA) at total 4 × 10 magnification in the samples to calculate density and biomass. Copepods and cladocerans were identified based on these papers [16, 17], and their wet weight was calculated according to the formula of these papers [18, 19]. Integrated water samples fixed with 1 mL saturated formaldehyde solution that was set volume to 50 mL was prepared for* Microcystis* spp. quantitative measurement. Colonial* Microcystis* was broken up to individual cells by an ultrasonic wave cell knapper (Model JY88-II, SCIENIZ, Ningbo, Zhejiang Prov., China) so that single cells could be counted. Fixed samples (0.1 mL) were tested using Olympus compound microscope under magnification of 40 × 10. Wet weight of* Microcystis* was calculated based on the formula of this paper [19]. All samples were collected at 7:00–8:30 a.m. to minimize variations between each sampling point.

Water chemistry including ammonia nitrogen (NH_4_^+^), nitrate nitrogen (NO_3_^−^), total nitrogen (TN), dissolved inorganic phosphorus, total dissolved nitrogen (TDN), total dissolved phosphorus (TDP), total phosphorus (TP), and chlorophyll-a were determined based on [20].* Microcystis* was measured at a sampling interval between two sampling points, and water chemistry was tested on every sampling point.

In this experiment, physicochemical water parameters, zooplankton, and* Microcystis* spp. data were collected. Regressions analysis among physiochemical parameters over the fish groups was undertaken to investigate interactions of each parameter.

### 2.4. Statistical Analysis

Data were normalized and variance was adjusted for homogeneity before analysis. Means of dissolved oxygen, pH, total dissolved solids, and transparency were compared between control group and each treatment during whole experiment using independent *T*-test. Chemical parameters and chlorophyll-a indicators with treatments and time as two factors were subjected to two-way ANOVA (analyze of variance) using post hoc multiple by comparisons test (LSD) [21] and expressed as means ± standard deviation (STDEV). Data for regression analyses were subjected to ln(*x* + 1) transformation. Differences were measured against control values and considered to be statistically significant at *P* < 0.05. Statistical analyses mentioned above were undertaken using SPSS (Statistical Product and Service Solutions, IBM Inc.) 13.0 for Windows. Statistical figures were output by R [22] and OriginPro 8.0 (OriginLab Corporation).

## 3. Results

### 3.1. Physicochemical Water Parameters

In this experiment, water temperature varied from 22.1°C to 26.2°C (lowest and highest values were recorded on 30th May and 3rd July). pH and total dissolved solids did not show significant different between control group and each treatment. MDG showed the lowest dissolved oxygen in the whole experiment. Higher fish density group presented lower dissolved oxygen. In the LDG, transparency was measured to be significantly higher than in the CG whereas the lowest occurred in the HDG and even lower than in the CG (Table 1).

### 3.2. Nutrients Change

**Figure 2:**
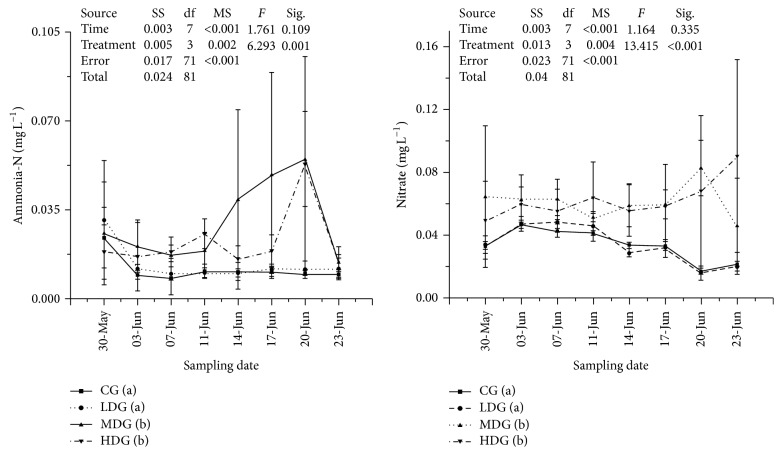
Nutrients values including ammonia and nitrate in each enclosure treatment during sampling points. Values were presented by mean ± STDEV. Treatments with different minuscules mean significant differences between each other and parameters in each treatment from low to high were arranged alphabetically (i.e., treatment with minuscule (a) means the lowest, at *P* < 0.05, *α* = 0.05). SS: type III sum of squares, df: freedom, and MS: mean square.

**Figure 3:**
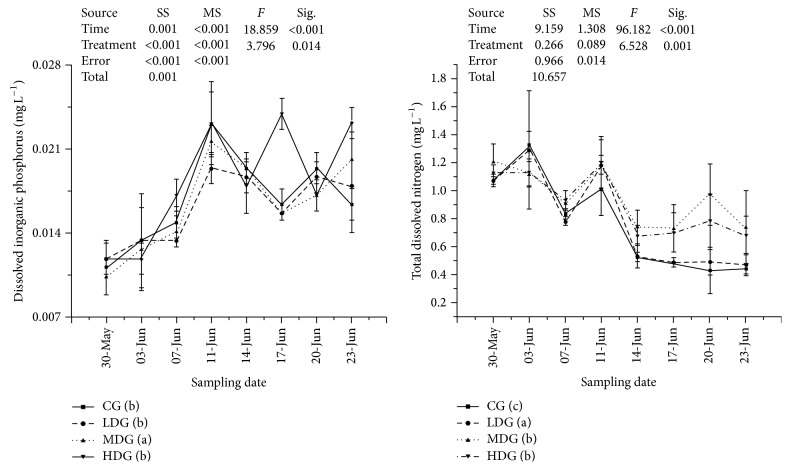
Nutrients values including dissolved inorganic phosphorus and total dissolved nitrogen in each enclosure treatment during sampling points. Values were presented by mean ± STDEV. Treatments with different minuscules mean significant differences between each other and parameters in each treatment from low to high were arranged alphabetically (i.e., treatment with minuscule (a) means the lowest, at *P* < 0.05, *α* = 0.05). SS: type III sum of squares, df: freedom, and MS: mean square.

**Figure 4:**
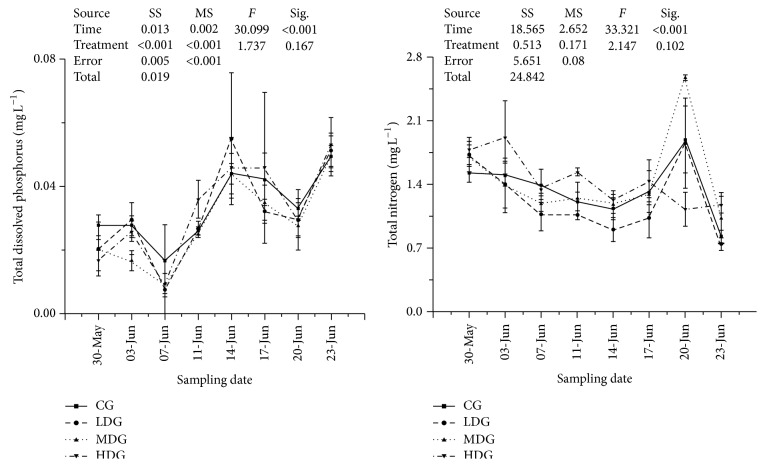
Nutrients values including total dissolved phosphorus and total nitrogen in each enclosure treatment during sampling points. Values were presented by mean ± STDEV. No significant differences between each other and parameters in each treatment. SS: type III sum of squares, df: freedom, and MS: mean square.

**Figure 5:**
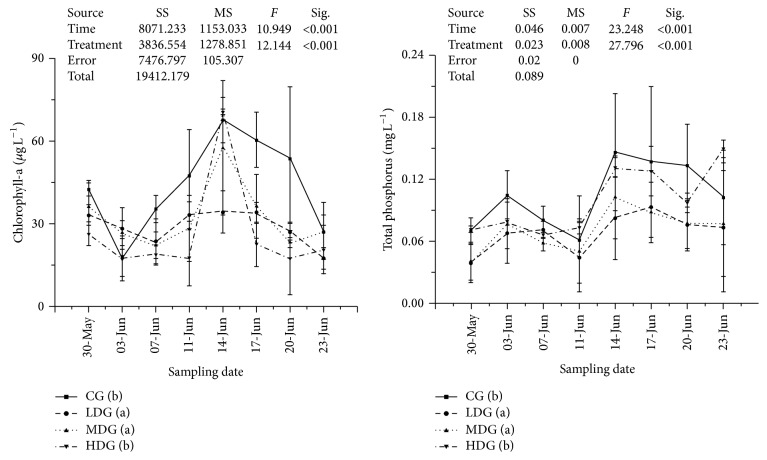
Nutrients values including total phosphorus and chlorophyll-a in each enclosure treatment during sampling points. Values were presented by mean ± STDEV. Treatments with different minuscules mean significant differences between each other and parameters in each treatment from low to high were arranged alphabetically (i.e., treatment with minuscule (a) means the lowest, at *P* < 0.05, *α* = 0.05). SS: type III sum of squares, df:freedom, and MS: mean square.

Nitrate and ammonia were found to be significantly higher (*P* < 0.05, df_7,3_, other statistics were shown in Figure 2) in MDG and HDG during this experiment. Dissolved inorganic phosphorus was lowest in MDG (Figure 3). Total dissolved nitrogen was lowest in LDG, increased with increasing fish biomass and highest in no fish group (Figure 3). None of differences were found between treatments in total dissolved phosphorus and total nitrogen (Figure 4). Chlorophyll-a and total phosphorus were lower in LDG and MDG that compared with control and HDG (Figure 5).

During this experiment, TN : TP ratio values were lowest in CG (Table 2). Significant difference of TN : TP value was found between CG and MDG (*P* = 0.028, *t* = −2.27). Regression analyzes in fish groups between chlorophyll-a to TN and TP showed that TP was positively related to chlorophyll-a (*P* < 0.001, *r* = 0.413) while TN was less relative (*P* = 0.095, *r* = 0.2) to chlorophyll-a fluctuate (Table 3). Regression analyzes showed that TN was positively related to TN : TP (*P* < 0.001, *r* = 0.856) while TP was not related to TN : TP (*P* = 0.514, *r* = −0.079).

Transparency (*Z*_Sd_) was negative related to* Microcystis* (*P* < 0.01, *r* = −0.47), TP (*P* < 0.01, *r* = −0.33), and chlorophyll-a (*P* = 0.01, *r* = −0.54). But TN was not observed related to all these parameters (Table 3).

### 3.3. *Microcystis* spp. and Crustaceans

Mean* Microcystis* during this experiment was lowest in the LDG while the highest was in the CG. The mean* Microcystis* biomass was significantly different in the fish groups to the control (Table 4). Dominant crustacean zooplanktons in our study were identified as* Limnoithona sinensis*,* Mesocyclops leuckarti*,* Thermocyclops taihokuensis, Bosmina *spp., and* Diaphanosoma *spp. Other zooplankton species were also found:* Ceriodaphnia cornuta*,* Sinocalanus dorrii*,* Thermocyclops *spp.,* Canthocamptus* spp., and* Moina micrura*. During this experiment, crustaceans decreased significantly in the fish enclosures (Table 4).

## 4. Discussion

In the present study, fish at the lower density (35 g m^−3^ to 70 g m^−3^) inhibited cyanobacteria blooms more efficiently. These enclosures performed as refined water quality, lower nutrient, and cyanobacteria density; relative higher zooplankton biomass than higher fish group.

The vital debates on successful biomanipulations in a long period usually depend on whether they can efficiently release internal load [23]. Closed system, just like in the present study, soluble nutrients, for example, NH_4_^+^ and NO_3_^−^, increased with increasing fish density, indicating density dependent effects that fish interfere water chemical process by their metabolism. Studies have illustrated TN, TP, and chlorophyll-a in the fish presence enclosures significantly lower than in the fish absence [24]. At relatively low densities, the silver carp were able to graze for particles of food directly, resulting in a decline in phosphorus and chlorophyll-a levels within the Lake Taihu enclosures [25]. Hence, the threshold of fish density usually should be taken into account for evaluating the risk and the advantage that fish could bring out. If the systems were enlarged to full-lake scale manipulation, the results are mixed at best.

The authors reported that reduction of stocking fish density promoted water quality in four Netherlands and Denmark shallow lakes [26]. Pond study also supported the result [27]. In the 1990s, an in situ enclosure experiment of silver carp manipulation was carried out in Lake Donghu, the experimental results showed that stocking density of 46–50 g m^−3^ silver carp can more effectively control the algae bloom, and the algae bloom was removed accompanied with decreased nutrients, which is similar to our experimental results [8]. Previous study proposed that water quality in pen stocked with about 40 g m^−3^ filter-feeding silver carp near our experiment area did not differ from outside water because of the large water exchange both inside and outside pen area [9]. The authors countered those 3 cases out of 11 filter-feeding fish biomanipulations in enclosure experiments which showed decreased total phosphorus, while 5 cases showed no effects and remaining 3 cases showed increased total phosphorus [28]. These evidences prove that filter feedings fish could interfere with water physicochemical process, so we want to study how the filter feedings fish could interfere with water physicochemical process.

In the present study, the increasing value of total nitrogen to total phosphorus ratio in fish presence enclosures indicated that cyanobacteria bloom was alleviated by fish, by referring cyanobacteria bloom explosion usually results in decline of TN : TP ratio [29]. In the present study, N did not decrease by fish's grazing, and consequently, increased TN : TP ratio in fish groups should be caused by TP decline. This means the top-down effects by fish to algal community was triggered by P fluctuation. Usually, P is considered to be the first regulatory factor that can limit growth of algae communities, while N is the secondary factor [30]. According to the positive relation between TP and chlorophyll-a in the present study, P decline promoted the possibility of limitation of algal growth from bottom-up, even though the absolute phosphorus (average TP > 150 *μ*g L^−1^) and nitrogen (average TN > 1 mg L^−1^) in the study area were enough for the cyanobacteria growth.

## 5. Conclusions

The present study provide evidences that, in enclosure conditions, fish at a density of 35 g m^−3^ to 70 g m^−3^ could be effective in controlling* Microcystis* blooms, promotion of fish production, and ameliorating the aquatic environment. Nonclassic biomanipulation is a proper means to reduce nutrients and phytoplankton under conditions of (1) eutrophic or hypereutrophic water, (2) lack of large sized zooplankton, and (3) dominance of filamentous or colonial algae.

## Figures and Tables

**Table 1:** pH, dissolved oxygen (DO), total dissolved solids (TDS), and Secchi depth (*Z*_Sd_) were measured during this experiment among each treatment. Significant differences compared between treatments and control groups were presented as means ± STDEV with different minuscules.

Parameters	Treatments	Means ± STDEV	Levene's test of variance of homogenous		*T*-test of means compared with control group		
			*F*	Sig.	*t*	df	*P* (two tails)
pH	CG	8.19 ± 0.42 (a)	—	—	—	—	—
	LDG	8.00 ± 0.40 (a)	0.506	0.481	1.602	46	0.116
	MDG	7.93 ± 0.36 (b)	1.508	0.226	2.284	46	0.027
	HDG	8.03 ± 0.39	0.400	0.530	1.319	46	0.194

DO (mg/L)	CG	7.72 ± 3.42 (a)	—	—	—	—	—
	LDG	6.21 ± 1.71 (a)	5.258	0.026	1.930	33.872$	0.062
	MDG	5.73 ± 2.23 (b)	2.099	0.154	2.384	46	0.021
	HDG	6.03 ± 2.17 (b)	2.129	0.151	2.036	46	0.048

TDS (g/L)	CG	0.42 ± 0.06	—	—	—	—	—
	LDG	0.45 ± 0.11	1.520	0.224	−1.004	46	0.321
	MDG	0.44 ± 0.10	1.010	0.320	−0.606	46	0.547
	HDG	0.40 ± 0.10	0.402	0.529	1.208	46	0.233

*Z*_Sd_ (cm)	CG	68.7 ± 9.9 (a)	—	—	—	—	—
	LDG	83.3 ± 12.7 (b)	0.088	0.768	−4.390	46	0.000
	MDG	75.2 ± 12.9 (b)	1.704	0.198	−1.950	46	0.057
	HDG	65.6 ± 12.7 (a)	0.856	0.360	0.956	46	0.344

$, variance was not equal.

**Table 2:** N : P ratio values and comparison between CG and treatments for each treatment. Results of TN : TP ratio are performed as mean ± STDEV; means with asterisk “^*∗*^” stand for significant difference (*T*-test).

Group	TN : TP ratio values	Test for equality of variances		*T*-test for equality of means		
		*F*	Sig.	*t*	df	Sig.
CG	12.43 ± 6.57	Compared with CG				
LDG	14.70 ± 7.58	0.670	0.418	−1.095	45	0.279
MDG	17.72 ± 9.31^*∗*^	2.230	0.134	−2.271	46	0.028
HDG	13.87 ± 7.71	1.908	1.908	−0.693	46	0.492

**Table 3:** Pearson (upper-right corner) and Spearman's rank (lower-left corner) correlation coefficients (*r*) between variables. TP: total phosphorus, TN: total nitrogen, NO_3_: nitrate, NH_4_: ammonia, MC: *Microcystis*, *Z*_Sd_: Secchi depth, and Chla: chlorophyll-a.

	TP	TN	NO_3_	NH_4_	MC	pH	*Z*_Sd_	Chla
TP		0.57	0.13	0.10	0.21	0.03	0.00	0.01
TN	−0.07		0.86	0.84	0.18	0.14	0.16	0.10
NO_3_	−0.49	0.21		0.01	0.17	0.45	0.63	0.05
NH_4_	0.03	−0.03	0.37		0.89	0.34	0.88	0.07
MC	0.54	−0.16	−0.10	0.40		0.01	0.00	0.32
pH	0.04	−0.29	0.31	0.30	0.36		0.76	0.44
*Z*_Sd_	−0.33	0.21	−0.22	−0.11	−0.47	−0.43		0.01
Chla	0.43	0.20	0.35	0.27	0.44	0.72	−0.54	

**Table 4:** Crustacean and *Microcystis *biomass for each treatment. Statistics are performed as mean ± STDEV; means with different minuscules stand for significant difference.

Variable	Treatments			
	CG	LDG	MDG	HDG
Crustacean biomass (*μ*g/L)	87.67 ± 23.50 (a)	12.34 ± 6.77 (b)	6.89 ± 4.34 (b)	7.03 ± 5.51 (b)
		*P* < 0.01	*P* < 0.01	*P* < 0.01

*Microcystis *spp. biomass (mg/L)	6.53 ± 2.44 (a)	2.55 ± 0.43 (c)	4.18 ± 0.37 (b)	3.94 ± 0.22 (b)
		*P* < 0.01	*P* = 0.042	*P* = 0.031
